# Biocompatible Thermoplastics in Additive Manufacturing of Bone Defect Fillers: State-of-the-Art and Future Prospects

**DOI:** 10.3390/ma18163723

**Published:** 2025-08-08

**Authors:** Dagmara Słota, Karina Niziołek, Edyta Kosińska, Julia Sadlik, Agnieszka Sobczak-Kupiec

**Affiliations:** 1 Department of Materials Engineering, Faculty of Materials Engineering and Physics, CUT Doctoral School, Cracow University of Technology, 37 Jana Pawła II Av., 31-864 Kraków, Poland; karina.niziolek@doktorant.pk.edu.pl (K.N.); edyta.kosinska@doktorant.pk.edu.pl (E.K.); julia.sadlik@doktorant.pk.edu.pl (J.S.); 2 Department of Materials Engineering, Faculty of Materials Engineering and Physics, Cracow University of Technology, 37 Jana Pawła II Av., 31-864 Kraków, Poland

**Keywords:** PEEK, PCL, PLA, PMMA, composites, bone, biomaterials, hydroxyapatite

## Abstract

The development of materials engineering allows for the creation of new materials intended for 3D printing, which has become a key tool in tissue engineering, particularly in bone tissue engineering, enabling the production of implants, defect fillers, and scaffolds tailored to the individual needs of patients. Among the wide range of available biomaterials, thermoplastic polymers such as polycaprolactone (PCL), polylactic acid (PLA), polyether ether ketone (PEEK), and polymethyl methacrylate (PMMA) are of significant interest due to their biocompatibility, processability, and variable degradation profiles. This review compiles the latest reports on the applications, advantages, limitations, and modifications in bone tissue engineering. It highlights that PCL and PLA are promising for temporary, resorbable scaffolds, while PEEK and PMMA are suitable for permanent or load-bearing implants. The inclusion of ceramic phases is frequently used to enhance bioactivity. A growing trend can be observed toward developing customized, multifunctional materials that support bone regeneration and biological integration. Despite ongoing progress, the biocompatibility and long-term safety of these materials still require further clinical validation.

## 1. Introduction

The continuous advancement in science, including automation, robotics, and materials engineering, has significantly contributed to the rapid development of additive manufacturing technologies, commonly known as 3D printing. This technology has revolutionized the field of biomaterials, particularly in tissue engineering. Among the many applications of 3D printing, bone defect reconstruction has emerged as one of the most promising and dynamically growing areas. Although bone tissue possesses natural regenerative capabilities, in the case of large, critical-size defects, medical intervention is necessary to restore both its structure and function [1,2]. Such defects can arise from trauma, tumors, infections, or congenital anomalies [3]. Traditionally, bone defect treatment and reconstruction have relied on autografts or allografts. An autograft involves harvesting bone from another area of the patient’s body, usually a region of low aesthetic or functional significance, and shaping it to fit the defect site. In contrast, allografts involve transplanting bone tissue from a donor, typically a deceased individual. However, both donor and recipient must have compatible antigens, and the recipient often requires immunosuppressive medication after the procedure. Immunological complications may also occur. Additionally, waiting for a suitable donor can be time-consuming, ranging from several months to even years. These types of grafts come with a number of limitations, such as the risk of complications at the donor site, immune response, and limited material availability [4,5,6,7]. As a result, 3D printing has emerged as a valuable alternative, offering the possibility of producing customized bone implants tailored to the individual anatomical and clinical needs of each patient [8]. This technology enables precise control over the geometry and internal structure of printed components, allowing for the creation of implants that perfectly match the size and shape of the defect [9].

Conventional techniques for producing biomaterials include solvent casting, particulate leaching, gas foaming, or freeze-drying. Compared to these, 3D printing provides greater and more precise control over scaffold geometry, porosity, and even internal architecture [10,11]. The risk associated with traditional manufacturing methods is often a problem with repeatability, both structurally and mechanically. In contrast, 3D printing allows complex, patient-specific implants to be manufactured layer by layer with controlled repeatability [12,13]. The materials can also be easily customized to the needs of a specific patient, requiring practically no post-processing. This high level of personalization is particularly important in bone tissue engineering, where size or shape varies greatly from patient to patient [14]. As such, the use of 3D printing provides a solution to many of the limitations associated with conventional techniques and thus offers great potential for personalised medicine.

Another advantage of 3D printing in bone tissue engineering is the wide selection of usable materials, especially thermoplastic polymers, which are known for their biocompatibility and ease of processing. Thermoplastics can be melted at elevated temperatures and solidified upon cooling, making them ideal for extrusion-based printing techniques such as fused deposition modeling (FDM). Within this material group, there are both biodegradable and non-degradable options, allowing for their selection depending on the specific clinical application. Some cases may only require temporary support for tissue regeneration, while others may need permanent structural replacement. Biodegradable materials offer the added benefit of gradually degrading and being replaced by natural bone over time, thus eliminating the need for implant removal.

Moreover, thermoplastics can be enhanced by incorporating bioactive additives such as calcium phosphate ceramics, which improve their osteoconductivity—defined as the ability of a material to serve as a scaffold that supports the attachment, migration, and growth of new bone cells—and promote better integration with surrounding bone tissue. This enables the fabrication of patient-specific implants that not only match the defect anatomically but also possess improved biological performance due to the presence of bioactive phases.

An important aspect is that, despite the great potential of 3D printing in bone tissue engineering, there are several challenges associated with this technology. The processing of thermoplastics requires precise control of temperature, printing speed, and post-processing conditions. Incorrect settings can lead to defects in the printed structure, such as warping, delamination, or incomplete bonding between layers [15,16]. Furthermore, repeatability between printers and print batches can be an issue, especially when progressing from laboratory-scale research to clinical-level production. The lack of standardised protocols for assessing the biocompatibility and long-term behaviour of printed constructs is also an issue [17,18].

This review aims to summarize the current state of knowledge regarding biocompatible thermoplastic polymers used in 3D printing for bone tissue applications. The following chapters focus on composite materials based on polycaprolactone (PCL), polylactic acid (PLA), polyether ether ketone (PEEK), and polymethyl methacrylate (PMMA), discussing their properties, modifications, and highlighting their potential in the development of modern regenerative materials.

## 2. Overview of Biocompatible Thermoplastic Materials

One of the key factors determining the application of 3D printing in a medical context is the selection of appropriate materials, which must not only meet mechanical and technological requirements, but also exhibit biocompatibility, meaning that they are biologically safe and do not cause a negative reaction from the human body [19]. In the context of bone tissue engineering (TE), biocompatibility is particularly critical, as implanted materials must support cell adhesion, proliferation, and differentiation, while integrating with the surrounding bone tissue without eliciting an immune response. Additionally, such materials should ideally promote osteoconductivity and, in some cases, osteoinductivity to facilitate effective bone regeneration [20]. Among the wide range of materials used in additive manufacturing, particular attention is given to thermoplastics, which, due to their processing properties and biological compatibility, are used in implantology, prosthetics, reconstructive surgery, and tissue engineering [21,22]. Biocompatible thermoplastics used in bone tissue engineering can be broadly divided into two main groups: bioresorbable polymers, such as polylactic acid (PLA) and polycaprolactone (PCL), and non-resorbable high-performance polymers, such as polyether ether ketone (PEEK) and polymethyl methacrylate (PMMA). These materials differ in terms of degradation rate, mechanical strength, and clinical application profile, offering diverse functionalities depending on the therapeutic strategy. 

**Figure 1:**
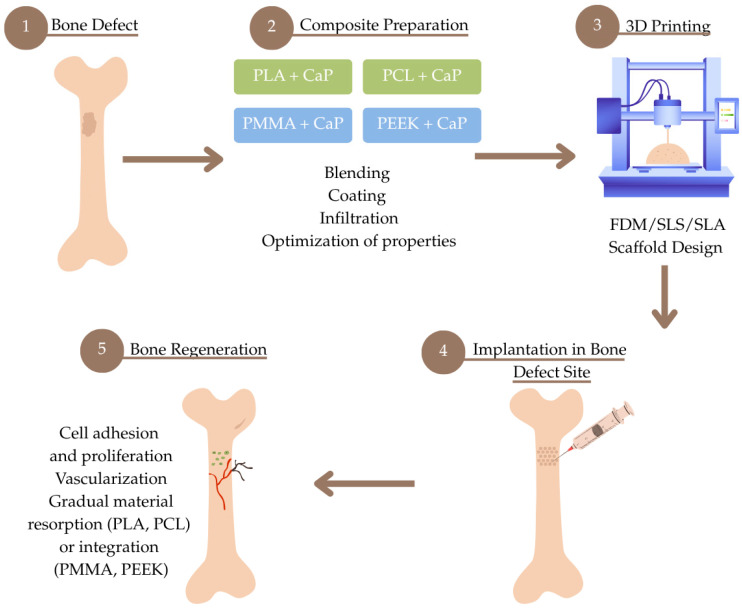
Schematic overview of the bone regeneration process using 3D printing and biocompatible thermoplastic composites.

This chapter presents an overview of selected biocompatible thermoplastic materials modified with a ceramic phase, which play a significant role in 3D printing. Their physicochemical properties and potential clinical applications are discussed. To provide a comprehensive overview of the discussed approaches, a schematic Figure 1 has been included at the beginning of the manuscript, illustrating the interplay between bone regeneration processes, 3D-printing technologies, and the thermoplastic materials described in this review. 

### 2.1. PEEK (Polyetheretherketone) in Bone Defect Bimaterials

**Figure 2:**
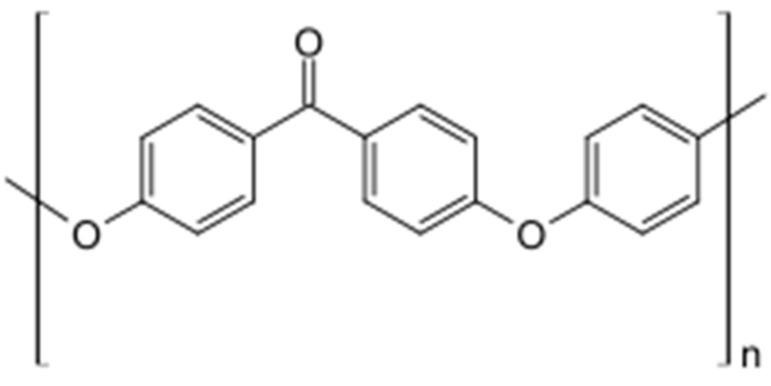
The chemical structural formula of PEEK.

Polyaryletherketones are a group of high-temperature thermoplastic polymers. In the context of biomedical materials used for bone implants, the most commonly used representative of this group is polyetheretherketone (PEEK). PEEK is a semi-crystalline thermoplastic polymer distinguished by its impressive mechanical properties, high-temperature resistance, and chemical stability [23]. Its chemical structure is presented in Figure 2. Due to its strength, this material is used in the production of demanding components such as medical implants, piston parts, bearings, pumps, electrical cable insulation, and compressor plate valves. It is suitable for applications requiring continuous operation at high temperatures, with a melting point of 343 °C.

Compared to conventional bone implant materials such as titanium (Ti), PEEK exhibits a modulus of elasticity of approximately 2–3 GPa, which is comparable to that of cranial bone (about 4 GPa for the frontal bone and about 5 GPa for the parietal bone), and significantly lower than that of titanium (102–110 GPa) [24,25,26]. A lower Young’s modulus helps to reduce stress shielding between the implant and the bone, which is a major cause of failure in Ti-based metallic implants. Moreover, PEEK has a tensile strength of 70 to 100 MPa and does not cause artifacts during radiographic imaging, computed tomography (CT), or magnetic resonance imaging (MRI), which is particularly important in postoperative assessment and represents another advantage over metal implants [27,28]. For these reasons, PEEK is widely investigated as a material for bone reconstruction. However, a significant limitation in its use, particularly in load-bearing applications, is its inability to form direct bone apposition and the resulting lack of osseointegration. This is attributed to the material’s bioinertness and, consequently, its poor adhesion to surrounding host tissues [29].

Due to PEEK’s high melting point, it is not as easy to process and as pleasant to work with as, for example, PLA. For this reason, many literature reports propose composites of PEEK and ceramics like hydroxyapatite (HAp), where the ceramic is only a bioactive coating and not a polymer reinforcement. A study was conducted in which an implant made of PEEK and an analogous one with a nanoHAp coating were compared. The cylindrical part of the implants was placed in the femur, with the cap resting on top of the cortical bone to facilitate implant placement. The animal model used was New Zealand rabbits. Although biosafety was confirmed, as well as the fact that the coated implant demonstrated better osseointegration, it was observed that a lot of implants fell out of the implant site and were not well fixed there. This is probably due to the fact that the polymer itself is inert and the coating is too thin to create a stable bond [30]. In another study, smooth, cylindrical PEEK implants were covered with a coating consisting of HAp and stabilized zirconium oxide (YSZ). Ion beam-assisted deposition technology was used for this purpose. After deposition, heat treatment by variable frequency microwave annealing was applied. Then, by observing the in vivo response of uncoated and coated PEEK implants in the lateral femoral condyle of rabbits, it was shown that the heat-treated materials exhibited better implant fixation, as well as greater bone regeneration and bone-to-implant contact area compared to uncoated PEEK. This may be related to an increase in the degree of crystallinity of HAp due to high temperature [31].

However, the biggest opportunity for personalized implants comes from 3D printing. Many works point to the advantages of printing with PEEK. And while it is customizable and can be produced by 3D printing, the product can vary slightly from manufacturer to manufacturer, which affects melting and solidification temperatures. Since it requires annealing to a very high temperature, the printing speed must be adjusted to ensure that the structure can form as intended and stabilize before applying an additional layer. Applying layers too quickly can cause bonding and deformation of the final product [32,33].

An interesting finding is the comparison of composites containing up to 30% ceramic 3D printed by Fused Filament Fabrication (FFF) compared to injection molded samples. It turns out that the FFF 3D-printing technique makes it possible to obtain materials with crystallinity recorded between 44.59% and 49.91%, typically higher than the values expected for injection molded samples. Such increased crystallinity significantly improves the mechanical properties of 3D printed materials. Here, the composite with 30% HAp showed a tensile strength of over 90 MPa, a value closely related to the performance of human femoral cortical bone [34]. It has also been shown that all 3D printed sample surfaces (from 5 to 30% ceramic) promote adhesion and growth of living U-2 OS osteoblast-like cells for up to 7 days [35]. The addition of the ceramic phase affects the extrusion parameters of the filament, and it was observed that as the proportion of ceramics increases, there is a need to increase the temperature. Moreover, the addition of 30% HAp in the composite significantly increases the melting point and crystallinity from 343 °C to 355 °C and from 27.7% to 34.6%. It also affects the hydrophilicity of the surface. In this study, HAp ceramics were modified with Sr or Zn ions, but no significant effect on the mechanical parameters of the final material was observed [36]. However, in another work where PEEK was modified with pure HAp and doped with Sr and Zn, an increase in Young’s modulus values was observed after the addition of pure ceramics and a decrease in values again under the addition of ceramics with ions [24]. Nevertheless, the mechanical parameters will depend not only on the % of ceramic, but also on the diameter and dispersion of its particles in the polymer. It has been shown that when the ceramic particles are uniformly distributed, changing their diameter has less of an effect on the elastic modulus. The modulus and anisotropic coefficient increased with increasing HAp content and particle clustering. Therefore, it is necessary to ensure particle size uniformity and dispersion homogeneity as much as possible to ensure the stability of mechanical properties at each location of the filament or printed sample [37].

However, the movement of the printer head, whether it prints horizontally or vertically, is not irrelevant when printing composite shapes. In studies where PEEK/HAp 30% composite was printed, as the ceramic content increased, the composite modulus increased, and the strength decreased. The lowest mechanical strength was observed for composites in the V-90° group, that is, printed vertically. The 30% HAp content caused an increase in the tensile modulus of the composite by 68.6% compared to pure PEEK printed along a horizontal 90° path, while the tensile strength decreased by 48.2% compared to pure PEEK printed along a vertical 90° path [38].

The structure of the final print is also important. In a comparative analysis of the print with PEEK, porous PEEK, and porous PEEK with HAp, it was found that cells proliferated better with porous materials. Histological examination and fluorescent staining showed that notably more new bone tissue was formed in the porous group with HAp. However, the porous structure significantly reduces the mechanical properties [39].

Also described are cases where the proportion of the ceramic phase was as high as 40%. It was shown that the elastic moduli of HAp/PEEK composites increased with increasing ceramic content in the material. However, on the other hand, this results in a loss of tensile strength and a decrease in ductility. Nevertheless, for composites with 30–40% ceramics, the elastic moduli were in the lower range of the elastic modulus of cortical bone and were 7–30 GPa, suggesting that they could find implant applications. This is also confirmed by in vivo results, which demonstrated higher osseointegration efficiency around the composite than around pure PEEK [40]. Similar findings were also confirmed in another study, where bioactivity was also confirmed during incubation in SBF, which resulted in the appearance of new apatite layers [41].

Given the above, PEEK and its ceramic composites offer promising possibilities for clinical application in bone tissue engineering, especially in the context of 3D printing. The material’s favourable mechanical properties and ability to be customised via additive manufacturing make it suitable for the production of tailored implants. The inclusion of ceramic phases, such as HAp, increases their bioactivity and osseointegration potential, making them a real alternative to metal implants in selected applications. Current research supports the use of 3D printed PEEK composites in craniofacial, orthopaedic, and spinal implants, indicating their growing clinical importance [25,42]. However, the issue of recycling PEEK is becoming increasingly important due to its high production costs and increasing use in medical and industrial applications. This polymer is not easy to recycle using traditional methods, as under standard processing conditions, it can degrade. However, there are strategies to enable its reuse. Mechanical recycling is possible through reprocessing, although it often leads to some loss of material properties. More promising is chemical recycling, which allows depolymerisation. The recovered substances can be used to re-synthesise PEEK or other products [43,44].

### 2.2. PCL (Polycaprolactone-Lactide) in Bone Defect Bimaterials

**Figure 3:**
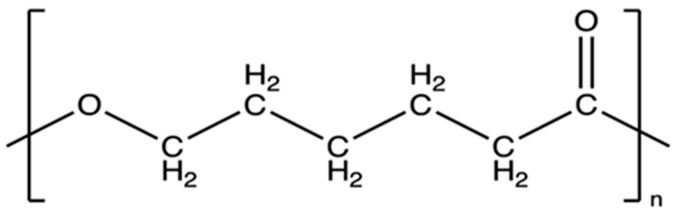
The chemical structural formula of PCL [54].

Poly(ε-caprolactone) (PCL) (Figure 3) is an aliphatic, biodegradable polyester obtained by ring polymerization of ε-caprolactone [45,46]. Its polymer chains contain regularly spaced ester groups, which give it a hydrophobic character, but at the same time allow hydrolytic breakdown of ester bonds in aqueous media [47]. The PCL degradation reaction proceeds mainly by non-enzymatic hydrolysis of these bonds, and the degradation products are then metabolized to 6-hydroxyhexanoic acid [48,49,50]. PCL is primarily synthesized via ring-opening polymerization (ROP) of the monomer ε-caprolactone, a cyclic ester with the molecular formula C_6_H_10_O_2_ [51]. The reaction conditions—such as the choice of catalyst, initiator, temperature, and reaction time—significantly influence the molecular weight, end-group functionality, and overall properties of the resulting polymer. The most commonly used catalyst is stannous octoate (Sn(Oct)_2_), favored for biomedical applications due to its low toxicity [52,53].

Polycaprolactone (PCL) has a relatively low melting point (about 58–63 °C) and a glass transition temperature of approximately −60 °C. These thermal properties result in significant flexibility and ductility of PCL under physiological conditions (~37 °C), making it suitable for biomedical applications. Its linear structure and low crystallinity promote good chemical processing and modification [55,56]. The long, linear chain structure makes it easy to form copolymers or functionalize polymer ends (e.g., by adding a carboxyl, amino, or hydroxyl group), which is of great importance in tissue engineering [57,58].

PCL has attracted increasing attention not only due to its biodegradability but also for its potential in various recycling strategies, including closed-loop chemical recycling. Several recent studies have demonstrated that PCL can be efficiently depolymerized into its monomeric or oligomeric forms and then repolymerized without significant loss of material properties. For instance, a zinc-catalyzed methanolysis process enables depolymerization of PCL into methyl 6-hydroxyhexanoate, which can subsequently undergo condensation polymerization to regenerate PCL, thus closing the material loop [59]. Similarly, thermal depolymerization catalyzed by stannous octanoate (Sn(Oct)_2) allows for highly efficient recovery of ε-caprolactone monomers (up to 98.1% conversion), which are then repolymerized into PCL with mechanical and thermal properties comparable to the original polymer [60]. In addition to monomer recovery, PCL can be chemically recycled into short-chain oligomers that act as efficient green plasticizers in PVC blends, improving flexibility and thermal stability, and demonstrating an upcycling pathway for post-consumer PCL waste [61]. Furthermore, embedding immobilized enzymes within the PCL matrix has been shown not only to accelerate its biodegradation but also to enable enzyme-assisted chemical recycling and subsequent repolymerization under mild conditions [62]. Together, these approaches present a versatile and sustainable framework for the recycling and revalorization of PCL-based materials.

Due to its beneficial chemical and mechanical properties, and because it is approved by the U.S. Food and Drug Administration (FDA), PCL is widely used in medical applications, particularly in bone tissue engineering [63]. Despite its favorable chemical properties, pure PCL exhibits low bioactivity—it does not contain functional groups that actively promote cell adhesion or induce mineralization [64,65]. For this reason, it is often combined with bioactive additives, such as hydroxyapatite, calcium β-triphosphate (β-TCP), or brushite, which improve its osteoconductive properties [66].

**Figure 4:**
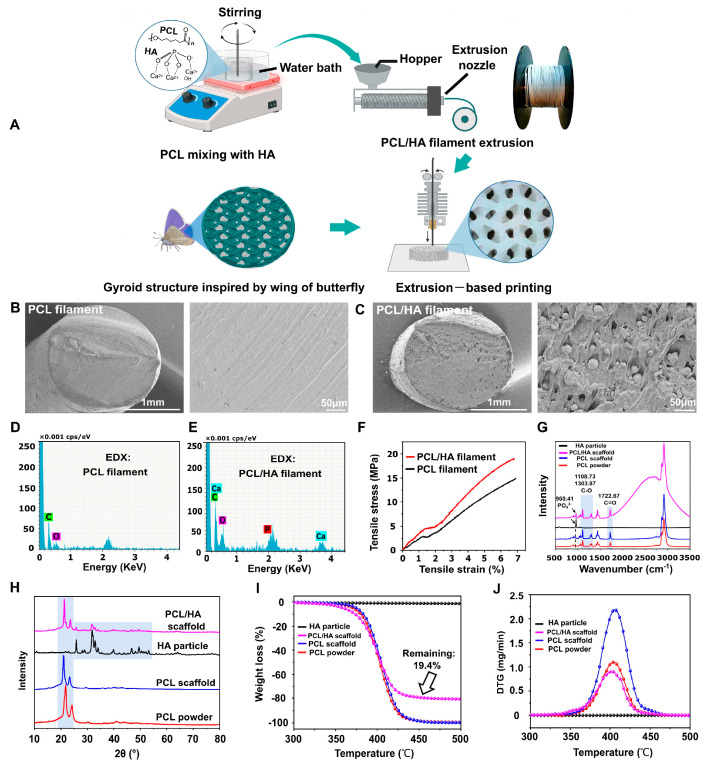
Filament manufacturing and printing of bionic scaffolds. (**A**) Illustration presenting the workflow for producing composite filaments and printing bionic scaffolds. (**B**,**C**) SEM images displaying cross-sectional views of pure PCL and PCL/HAp composite filaments. (**D**,**E**) Elemental mapping of the filament cross-sections to assess material distribution. (**F**) Tensile stress–strain curves comparing mechanical properties of PCL and PCL/HAp filaments. (**G**) Raman spectroscopy results of HAp particles, PCL/HAp scaffolds, pure PCL scaffolds, and PCL powder. (**H**) X-ray diffraction patterns highlighting the crystalline structures of HAp, PCL/HAp scaffolds, PCL scaffolds, and PCL powders. (**I**,**J**) Thermogravimetric analysis and derivative thermogravimetry profiles for HAp, PCL/HAp composites, PCL scaffolds, and raw PCL material [68].

The use of hydroxyapatite, especially of biological or synthetic origin, allows for increased bioactivity and mechanical stability of scaffolds, while enabling the design of porous structures similar to the architecture of natural bone. One study used about 15% by weight of nanometric HAp, extracted from biological waste such as clam shells, cuttlefish bones, or eggshells, in combination with PCL (85%) to 3D print porous scaffolds. The results showed that the presence of HAp significantly improved the mechanical properties of the scaffolds—the elastic modulus was within the range typical of barrel-shaped bone tissue (177–316 MPa). Importantly, increased adhesion, viability, and proliferation of MG63 cells were observed compared to pure PCL scaffolds, and the best biological results were obtained when HAp derived from mussel shells was used, indicating the influence of the biological source on the cellular response [67]. Another study focused on optimizing the structure of PCL/HAp scaffolds using gyroid geometry, as illustrated in Figure 4. Using the FDM method, scaffolds with varying degrees of filling (40–60%) were developed with constant HAp content. The best mechanical and hydrophobic properties were exhibited by the 55%-filled variant, which had pores with an average size of 465 ± 63 μm. This structure enabled not only good cell support but also high alkaline phosphatase activity and significant calcium salt deposition, confirming the osteogenic potential of such designed scaffolds [68]. In an effort to increase the HAp content of the composite, subsequent studies have shown that it is possible to obtain scaffolds with a very high ceramic phase content—up to 90% HAp by weight—while maintaining the material’s flexibility (elongation at break >100%) through the addition of poly(ethyleneglycol) (PEG). Importantly, the greater exposure of HAp particles on the scaffold surface contributed to more intensive extracellular matrix formation, although this did not translate into proportionally greater volume of newly formed bone after 12 weeks of implantation in rats. Regeneration occurred via intramembranous ossification, and the type of tissue formed (bony vs. fibrous) was not directly dependent on the degree of pore vascularization. The presence of immune cells, such as giant cells, suggests an important role of the inflammatory microenvironment in regenerative processes [69]. Another approach focused on Voronoi architecture, a modern structural design scheme used in advanced 3D-printing technologies. The study used a composite containing only 4% HAp,. The scaffolds had high porosity (73%) and very low degradation (<1% by weight after 6 months in vitro). When the scaffolds were loaded with fresh xenograft material and implanted in animals, intense angiogenesis, absence of fibrous capsule, and endochondral bone formation were observed, confirming the clinical potential of Voronoi scaffolds in the context of scaffold-guided bone regeneration (SGBR) [70]. In addition, there is growing interest in multi-component composites containing not only PCL and HAp, but also biopolymers such as chitosan (CS), which exhibits antimicrobial properties and promotes tissue regeneration [71,72]. One study developed scaffolds containing 30% HAp, 5% CS, and PCL, showing very good hydrophilicity, confirmed by wetting angle measurements, and high bioactivity. These scaffolds effectively reduced inflammation due to CS, and their structure promoted cell proliferation and bone tissue regeneration both in vitro and in vivo. Moreover, higher HAp content (30%) correlated with better regeneration results compared to scaffolds containing only 10% HAp. However, the presence of fibrous connective tissue and residual material in some areas indicated a complex healing process, depending on the local conditions of the implant environment [73].

In recent years, PCL and β-TCP composites have also been intensively studied as bone regeneration materials. In the study, Niu et al. used 3D-printing technology to obtain PCL/β-TCP lattice structures with a similar structure to natural bone. The results showed that these structures, especially the PT2 variant, had better mechanical and hydrophilic properties, and promoted MC3T3-E1 cell adhesion and proliferation, as well as the formation of new bone tissue [74]. Increasing the proportion of β-TCP in the composite results in an increase in stiffness (Young’s modulus), hydrophilicity, and surface roughness, which translates into better adhesion and differentiation of osteoblasts. The authors also noted the possibility of producing structures with up to 70% β-TCP content without the use of solvents [75]. Helaehil et al. evaluated the effect of bioelectric stimulation (ES) combined with HAp/β-TCP scaffolds on bone defect regeneration. They found an increase in BMP-7 expression and angiogenesis, as well as beneficial modulation of the TGF-β/BMP pathway and RANKL/OPG balance, which supported bone homeostasis [76]. Moreover, PCL/β-TCP scaffolds can be enriched with carbon nanotubes (CNTs), yielding a material with >60% porosity, suitable mechanical properties, and non-toxic performance, which promoted ADSC cell proliferation in vitro—especially when containing 0.2% CNTs [77]. Furthermore, an innovative PCL/β-TCP scaffold consisting of thick FDM-printed fibers and a microgrid of 10 μm fibers obtained by the MEW method was developed. This structure allowed cells to grow not only on the surface of the fibers, but also in their pores, resulting in better osteogenesis (ALP expression, mineralization) [78]. In contrast, the work of Lee et al. used an advanced therapeutic approach by combining a printed PCL/β-TCP scaffold with a polydopamine (PDA) coating and alginate microspheres (AM) containing BMP-2. This combination provided controlled release of BMP-2 factor and effectively promoted mineralization, expression of osteogenic markers, and regeneration of segmental femoral defects in rabbits [79]. All these studies demonstrate that PCL/β-TCP composites exhibit excellent mechanical properties, biocompatibility, osteoconductivity, and great potential in regenerating bone defects—both in vitro and in vivo applications.

The incorporation of calcium-based ceramics such as HA or β-TCP into PCL significantly enhances its mechanical properties and bioactivity. These fillers increase stiffness and hardness, while also promoting apatite formation and bone integration, which are lacking in neat PCL [80]. However, processing challenges arise, including poor filler dispersion, reduced flowability, and weak interfacial adhesion. High ceramic content may also lead to brittleness. Surface modification of the ceramic particles and optimization of processing conditions are essential to achieve uniform composites with reliable performance [81].

PCL with the addition of bioactive ceramic materials such as hydroxyapatite, tricalcium phosphate, or bioactive glass (BGS-7) is gaining increasing importance in clinical bone regeneration due to their osteoconductive properties, biocompatibility, and ability to be personalized with 3D printing. Approaches combining such scaffolds with mesenchymal stem cells (MSCs) and growth factors, such as FGF-2 and BMP-2, are particularly promising. In preclinical studies, MSCs populated on nHAP-coated PCL/HAP/β-TCP scaffolds, after prior stimulation with FGF-2 and BMP-2, have been demonstrated to exhibit significantly higher adhesion, proliferation, and osteogenic differentiation. After implantation of such constructs in the ovine mandibular region, induction of bone regeneration and good integration with the surrounding tissue without inflammatory reaction were observed [82]. The clinical potential of these solutions is also confirmed by a study involving patients with craniofacial defects, in which 3D printed PCL/BGS-7 implants were used. This material presented apatite-forming ability in vitro and good stability and precision of reconstruction in vivo, with minimal complications [83]. These results indicate that PCL/ceramic composite scaffolds, especially when combined with MSCs and osteogenic agents, may find wide clinical application in the treatment of extensive bone defects, including in orthopedics, maxillofacial surgery, and neurosurgery.

### 2.3. PLA (Polylactic Acid) in Bone Defect Bimaterials

Polylactic acid, polylactide, or known by the acronym PLA, is a very versatile biopolymer. An important function it has is biodegradability and thermoplasticity. Its renewable origin, as well as its favourable mechanical properties, have made PLA one of the most popular polymers used in modern materials engineering. Polylactic acid has modern industrial applications, mainly textiles and packaging, but this is not its only role [84,85,86]. Following the development of PLA, in the 1960s, studies began for the use of this polymer in surgical implants, as well as tissue repair and other biomedical applications. The polymer can be found in fields such as tissue engineering, dentistry, and cardiology. The properties that post-l-lactic acid possesses allow easy processing, rapid prototyping, and the creation of 3D materials.

Such a specification can be helpful in creating personalized implants or scaffolds used in tissue engineering. Also, one should not forget the possibility of producing medical equipment that provides protection for medical personnel [84,87]. It should be added that PLA belongs to shape memory polymers (SMPs), which differ from metallic alloys with shape memory, primarily due to their higher recoverable strain. Polylactide is a thermoplastic SMP that is of particular interest when it comes to applications in the medical industry due to features such as its high elastic modulus, relatively low Tg (55–65 °C), and the fact that it can be used for 3D printing. In addition, the shape memory properties of the polymer in question can be improved through various modifications, i.e., chopping, chemical modification, as well as the addition of various additives [88,89]. One solution is also the addition of inorganic particles to the PLA matrix, which can provide an additional solid phase. For materials used in bone tissue, calcium phosphate particles are very popular [90,91].

PLA, compared to other polymers, is a material with a high production capacity and, additionally, has a high yield. Moreover, this bioresorbable polymer is a promising product since the monomers can be produced from a non-toxic, renewable raw material, plus a naturally occurring organic acid [86]. The building block of polylactic acid is lactic acid, which is a chiral particle, and moreover, it can be produced by sugar fermentation from renewable sources, i.e., sugarcane or corn. For this reason, PLA is an environmentally friendly product, which translates into a better possibility of use in the human body [92,93] PLA is classified as safe (GRAS) by the United State Food and Drug Administration (FDA—inventory of effective food contact substance (FCS) notifications no. 178) and is safe for food packaging applications. The first synthesis of PLA was conducted in 1932 by Wallace Carother at DuPont Laboratories. At that time, he was able to obtain PLA of low molecular weight by heating lactic acid in a vacuum and removing condensed water. At that point, the biggest problem was to increase the molecular weight of the product and open the ring [94]. Currently, the polymer in question can be obtained by a number of methods. Each of them requires appropriate conditions, i.e., temperature, pressure, or pH, the use of catalysts, and a long polymerization time [95]. Various polymerization processes from lactic acid are used to obtain PLA, i.e., polycondensation, ring opening polymerization, as well as enzymatic polymerization and many others. The more popular and most widely used method is ring-opening polymerization [96]. It should be noted that PLA production, compared to other biopolymers, is distinguished by a number of advantages. One of them is environmentally friendly production, the product of which is a bioresorbable, recyclable, and compostable polymer. Biocompatibility is another fantastic property of the material. It is essential from the point of view of medical applications. A biocompatible material should not produce toxic or carcinogenic substances in tissues. It should also be emphasized that degradation products should not affect tissue healing. PLA degrades to its constituent α-hydroxy acid when implanted into a living organism, is sequentially incorporated into the tricarboxylic acid cycle, and excreted. Moreover, the degradation products of this polymer are safe, such that they are ideal for medical applications. The FDA has also approved PLA in direct contact with biological fluids [86,97,98].

An important advantage of PLA is its good thermal processability, which is much better compared to other polymers and allows processing by injection molding, film extrusion, blow molding, thermoforming, fiber spinning, and film forming [99].

Production is energy-efficient, with PLA requiring up to 55% less energy compared to petroleum-based polymers. In addition to the presented advantages, PLA also shows some disadvantages, i.e., poor strength. This limits the use of this polymer in applications that require plastic deformation at high stresses [100].

**Figure 5:**
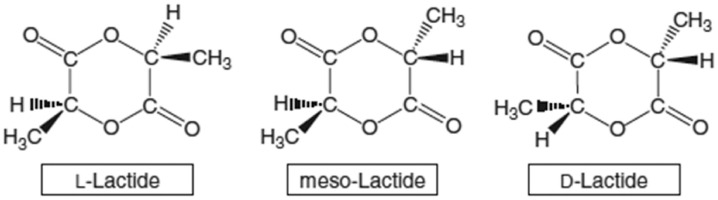
Illustrations of the chemical structures of L-, meso-, and D-lactides [85].

The mechanical properties of PLA can vary significantly depending on the molecular weight of the polymer and the degree of crystallinity. Due to the chirality of PLA monomers, it is possible to manipulate the mechanical properties by polarizing D-lactide, L-lactide, D, L-lactide, or meso-lactide. Figure 5 shows the chemical structures of L-, meso-, and D-lactides. It is also possible to maneuver the molecular weight through modifications, i.e., the addition of different functional groups hydroxyl species, lactic acid, and water). With the help of various modifications of the polymer backbone, the desired properties of PLA can be obtained [84]. PLA, as well as its copolymers, is compatible with living tissue. The limitation in this case is the L stereoisomer of PLA due to an enzyme produced by mammals, which leads to the degradation of this stereoisomer. The polymer in question is used in the production of screws or scaffolds, which are used as a temporary structure for adequate bone tissue growth. The component then breaks down after a certain period of time. A field that often uses PLA is orthopedic surgery, where artificial bones and joints are used, as well as surgical sutures.

PLA-HAp composites are increasingly popular for medical applications. The combination is a combination of the osteointegration capabilities that the bioactive ceramic brings to the system and the ease of processing of the polymer. For this reason, composites of this type have application potential in bone tissue [101]. The addition of bioactive ceramics, i.e., HAp, β-TCP, or bioactive glass (BG), can significantly affect the mechanical properties of PLA composites. On the other hand, hydroxyapatite, whose chemical structure is similar to the mineral phase of bone, promotes osteoconductivity as well as proliferation of osteoblasts [101,102]. Studies indicate that PLA/HAp composites have improved mechanical properties, including tensile strength, as well as better biological properties compared to the polymer alone. The addition of ceramics is an important element, as it can contribute to the formation of hydroxyapatite on the surface during in vitro tests, which translates into bone tissue regeneration [103]. Blending the polymer with bioactive materials, including ceramics, has also been shown to highlight benefits, combining the flexibility and processability of PLA with the bioactive advantages of ceramics [102]. PLA/HAp scaffolds have improved mechanical properties, making them more suitable for nasal applications in orthopedics [104,105]. PLA/HAp composites, due to their effectiveness in mimicking the properties of natural bone, are a promising material for use as scaffolds in regenerative medicine [106].

Ion Tcacencu et al. have developed a novel apatite–wollastonite/polylactic acid (AW/PLA) composite structure that matches the properties of cortical and spongy bone. The composite was produced using an innovative combination of 3D printed polymer and ceramic structures. They were joined together in a thermal manner. Biological tests performed indicated the biocompatibility of each of the material’s components, as well as the composite as a whole. The ceramic part supported the proliferation and differentiation of bone cells in the rat. In a calf defect model in the rat, the AW material was found to have an excellent effect on osteointegration, which translated into new bone formation and vascularization of the porous structure. This observation was noted both for the stand-alone structure and when it was part of the AW/PLA composite. The polymer–ceramic composite structure showed the largest amount of newly formed bone; this result is considered the presence of an AW structure [106].

Additive methods can be used to create custom medical products, such as synthetic bone models. Dan Wu et al. present a study in which the FDM method was used to produce a bone model that would better mimic real bone in terms of mechanical as well as biological properties. Therefore, a composite of degradable polymer PLA and HAp was used as a filament. Filaments with three compositions differing in hydroxyapatite content (5%, 10%, 15%) were evaluated. Microcomputed tomography of the printed bone models confirmed that the basic barrel structures were obtained. It was noted that the addition of ceramics reduced the accuracy of printing with respect to morphology, but an improvement in mechanical properties was observed. The mechanical properties of both the filament itself and the bead models were closer to those of human bone than the synthetic foams used [107].

Other research points to the use of ceramics in the form of BG for 3D printing. Nicolas Söhling et al. developed a 3D printable, osteoconductive scaffold system. They used PLA polymer and bioactive glass of 5%, 10% and 20%. Bioassays were conducted on mesenchymal stem cells (MSCs) and indicated that the materials produced were biocompatible. The presence of bioactive glass enhanced the bioactivity. However, the addition of bio-glass was found to have little effect on the expression of osteogenic genes, but had a significant effect on the expression of inflammatory genes in MSCs [108].

Other studies point to the use of composite materials containing a mixture of polymer and ceramics to accelerate bone tissue regeneration [109]. In such composites, polymers usually act as a matrix [110,111]. Gerardo Figueroa Romero et al., in their work, focused on the use of ceramics due to their osteoconductive properties. Many studies conducted indicate that the addition of ceramics to the PLA matrix can improve mechanical properties, cellular activity, and the bone formation process in general [112,113,114].

**Figure 6:**
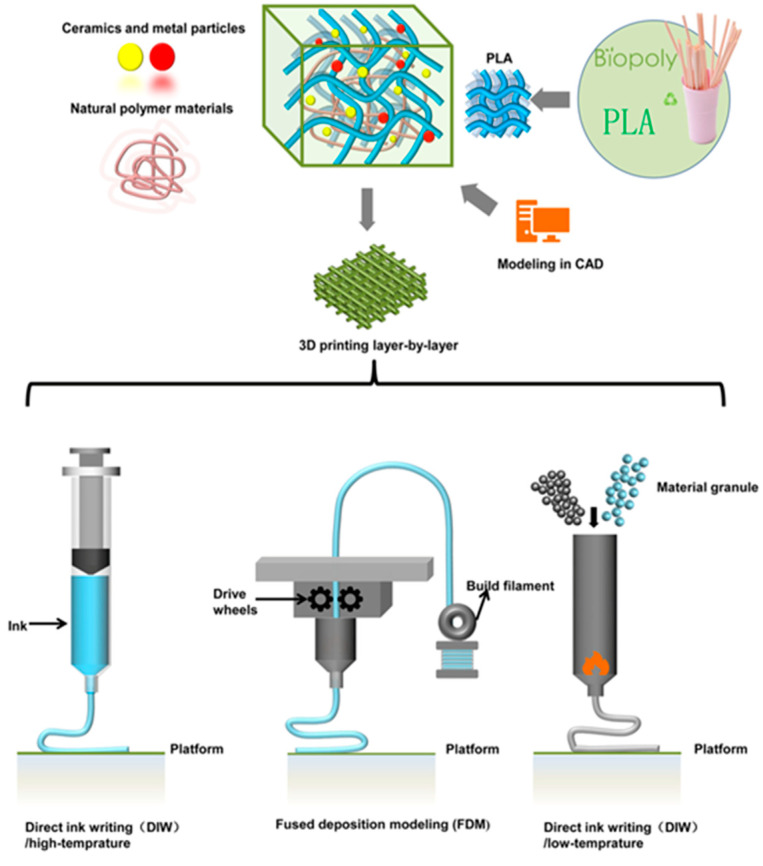
3D manufacturing process of PLA-based scaffolds.

Additive techniques often require the use of solvates, i.e., dichloromethane, which has been shown to be carcinogenic. The use of such reagents is quite controversial in implantology, as it can be dangerous to both human health and the environment [115,116,117]. In their study, Gerardo Figueroa Romero et al. used TCP as the ceramic, and PLA/TCP composite scaffolds were fabricated without the use of solvents. The study indicated that a higher ceramic overvalue results in better bone tissue regeneration. However, too high a TCP content, i.e., above 30%, reduces the flow rate and nozzle blockage during the printing process, which reduces process efficiency. The scaffolds were fabricated using the FDM method [118]. A number of studies conducted at the turn of the century indicate that for PLA/ceramic composites, the most popular applications are sutures, repair of fractures of the orbital floor, implants, drug-eluting stents, and drug delivery. The applications presented can be produced in many ways. One of the most popular is, of course, 3D printing, but also such methods as extrusion and injection molding, electrospinning, and solvent casting [90,119,120,121,122]. Figure 6 shows the 3D manufacturing process of PLA-based scaffolds [123].

The addition of inorganic fillers, in the form of HAp or other inorganic additives, helps to overcome the mechanical and biological limitations of PLA. The addition of hydroxyapatite improves the stiffness and strength of PLA due to better interfacial adhesion and the stiffening effect of the matrix. In the case of HAp particles with an ordered structure, the improvement in fracture resistance and durability is more beneficial due to the anchoring of the matrix in PLA. One of the most important advantages that hydroxyapatite fillers bring to the system is bioactivity. This translates into increased hydrophilicity of the material, which promotes cell adhesion and proliferation, making the material attractive in tissue engineering. However, there are several disadvantages to introducing fillers into the polymer, mainly related to technological problems. Particle agglomeration, increased melt viscosity, and reduced fluidity make it difficult to process the material, i.e., injection molding and 3D printing, and can also lead to structural defects. It should be emphasized that too high a content of hydroxyapatite additive can affect the brittleness of the material and cause uneven shrinkage during cooling. It has been found that the introduction of fillers can have positive effects on the manufactured material, but this requires precise control of the type of filler, its morphology, and its quantity [124,125,126].

An important aspect to note when discussing this polymer is its recycling. PLA, which is a biopolymer derived from renewable raw materials like corn starch, has favorable biodegradable characteristics, but its decomposition in the environment is slow. Therefore, a key way to extend the life cycle of PLA is to recycle it—especially chemical recycling. This process allows not only to recover monomers—lactic acid or other valuable chemicals, but also to maintain the quality of the material at a level close to the original—unlike mechanical recycling, which deteriorates the properties of the polymer. Moreover, chemical recycling of PLA is more tolerant of contamination and can be efficiently carried out using various methods such as hydrolysis, alcoholysis, or pyrolysis, creating a real opportunity to integrate PLA into a closed-loop economy [127,128,129,130,131].

PLA and its composites with ceramics are among the most promising materials used in 3D-printing technology for bone tissue regeneration. In addition, advances in 3D printing and the development of materials engineering are leading to effective solutions such as coat scaffolds that can simulate the natural bone structure and environment. PLA is distinguished by its biocompatibility, biodegradability, and good mechanical properties, which are comparable to spongy bone. Its easy processability with FDM technology allows the creation of personalized porous scaffolds that promote cell adhesion, proliferation, and osteogenic differentiation. In addition, PLA can act as a carrier for drugs or growth factors, broadening its potential clinical applications—from tissue engineering to orthopedics and dentistry. PLA composites with the addition of bioactive ceramics, such as HAp or β-TCP, show even greater potential. Such materials show much better osteoconductivity, mechanical strength, and the ability to form new bone tissue in large defects. In addition, the presence of ceramics allows for regulating the rate of degradation and increases the wettability of the surface, which improves the interaction with cells. PLA/ceramic composites are also used as personalized implants in craniofacial surgery and as drug carriers for the treatment of bone infections. The use of these materials in clinical bone regeneration opens new perspectives for personalized regenerative medicine. Current research indicates the great potential of these materials for use in additive methods [123,132].

### 2.4. PMMA (Polymethyl Methacrylate) in Bone Defect Bimaterials

Polymethyl Methacrylate (PMMA—IUPAC name: poly[1-(methoxy carbo-nyl)-1-methyl ethylene]) was first discovered in 1843 by Redtenbacher. He was the first to use this name and describe its properties [133,134]. However, since the 1940s, polymer has been used as an essential biomaterial in clinics and dental laboratories [135]. About a dozen years later, it was used in orthopaedic surgery on the femur for cementing [136].

It has found application in many fields, ranging from medical equipment, dental implants, drug delivery, and optics [134].

**Figure 7:**
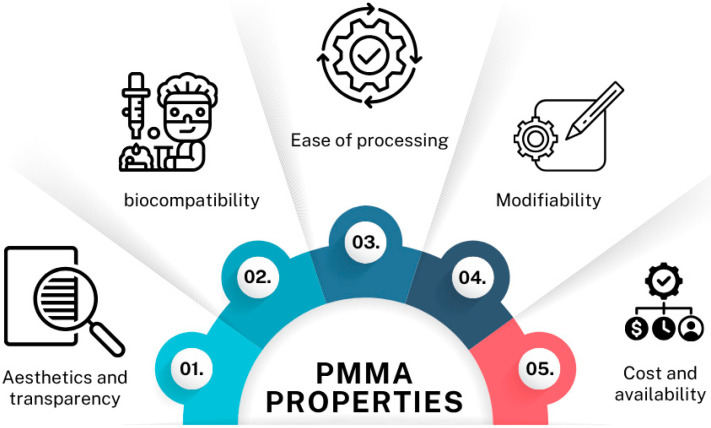
PMMA properties.

This polymer has gained interest as a biomaterial due to its ease of processing, aesthetics, cost-effectiveness, and satisfactory mechanical properties. Furthermore, it has low density and exceptional transparency [137]. Its properties are presented in Figure 7. This material is not sensitive to UV radiation, but when exposed to it, it may become discolored and its mechanical properties may deteriorate, leading to the formation of cracks. As a result of photodegradation, bonds are broken, leading to the formation of shorter polymer chains [138].

It is obtained by the addition of free radicals and the polymerization of methyl methacrylate to methyl polyacrylate [139].

In order for the process to take place, it is necessary to supply energy, such as microwaves, heat, or, as a result of a chemical process, because this will initiate and activate free radicals.

**Figure 8:**
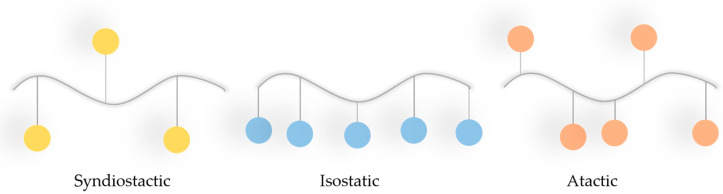
Different tacticities of PMMA (blue dots = ester groups of the PMMA).

PMMA belongs to the acrylate family and, considering its thermal properties, its glass transition temperature ranges from 100 to 130 °C, and its density at room temperature is 1.20 g/cm^3^. It is characterised by linear moulding shrinkage of 0.003 to 0.0065 cm/cm [140]. PMMA has good mechanical properties, i.e., high Young’s modulus and hardness (Table 1). Considering the stoichiometry of the bonds, this polymer can occur in isotactic, syndiotactic, and atactic forms (Figure 8) [139,141].

Depending on the tactical approach adopted by the polymer, its amorphous character will be as follows: syndiotactic > atactic > isotactic.

Due to its properties described above, PMMA has a wide range of applications in science and potential in various areas of technology. However, 3D-printed biomaterials are a rapidly developing industry. The integration of PMMA with ceramic materials in 3D-printing technology allows for the creation of structures with increased bioactivity and improved mechanical properties. Four examples of such composites are presented below.

Researcher E. Marin and her team set themselves the goal of developing a PMMA-based composite with added ceramics (AlN, BaTiO_3_, TiO_2_) that could be 3D printed using stereolithography (SLA) and would exhibit antibacterial properties. The composites consisted of PMMA and various concentrations of ceramic powders. The printing method used a laser to cure the liquid resin layer by layer, creating precise, complex three-dimensional models, i.e., stereolithography (SLA). The research showed that composites with the addition of aluminium nitride (ALN) had the best properties and demonstrated a strong effect against E. coli (up to 70% reduction in colonies). The addition of AlN (up to 15%) caused a moderate decrease in tensile strength (approx. 12%) and a reduction in deformation at break, but increased the modulus of elasticity. Synergistic properties were observed: good printability, preserved mechanical properties, and high antimicrobial efficacy. Although PMMA is bioinert, AlN functionalisation gives it bactericidal properties, which are important for bone defect fillings. Such a composite can be used as a temporary implant material or protective bone cement [145].

Chen et al. focused their attention on PMMA and the use of nanofillers to improve light-curing resins in order to enhance their mechanical and antibacterial properties. In these studies, they used TiO_2_ and PEEK as micron fillers. Nano-TiO_2_ is a long-acting antibacterial agent with beneficial photocatalytic properties. Under UV radiation, it can produce reactive oxygen species that effectively destroy bacteria [146]. The studies showed that PMMA composite resins enriched with 1% TiO_2_ and 1% PEEK had the most favourable properties among all the groups analysed. The addition of 1% TiO_2_ nanoparticles effectively improved both the mechanical properties and the antibacterial activity of the material. In addition, the addition of TiO_2_ to the composite resin showed excellent antibacterial properties compared to pure PMMA resin. Furthermore, a model made from this resin suggests that such a functional material may be a suitable light-curing resin for use in 3D printing in dentistry [147].

Other reports mention potential materials for filling bone defects based on PMMA and hydroxyapatite. HAp in the form of pure powder exhibits bioactivity and good biocompatibility, but due to its properties, it is difficult to give it the precise shape required for bone tissue reconstruction. In the presented study, Tontowi et al. combined HAp with PMMA to create a biocomposite that can be used as a feedstock for 3D printers in biomedical applications. In this case, it was important to achieve a balance between short curing time and adequate tensile strength. Although the addition of HA could lead to a weakening of the material’s mechanical properties, its use was intended to increase bioactivity and the ability to conduct bone tissue growth (osteoconductivity), as well as to accelerate the curing process, which was key to improving and speeding up the printing process [148].

In their work, Esmi et al. developed an innovative PMMA-based nanocomposite reinforced with multi-walled carbon nanotubes (MWCNT) and nanohydroxyapatite (nHAp) for 3D printing [149]. The composite was obtained by melt mixing and then converted into filaments for FDM (Fused Deposition Modelling) technology. The authors used 0.1% wt. CNT and up to 15% wt. HAp, with the best mechanical properties obtained for a composition of 0.1% CNT + 4% HAp—a 375% increase in elastic modulus and more than a twofold increase in hardness compared to pure PMMA (based on nanoindentation analysis) were demonstrated. The presence of MWCNT had a positive effect on the dispersion of HAp particles in the polymer matrix, which was attributed to interfacial interactions, including ion-dipole effects between the polar groups of HAp and the aromatic surface of the nanotubes. Rheological and morphological analyses confirmed the homogeneous distribution of nanoparticles for HAp contents up to 6% by weight. Cytotoxicity studies conducted using L929 fibroblasts showed that the composite containing both CNT and HAp promoted cell adhesion, proliferation, and survival. Although CNTs alone showed toxic potential, their combination with HAp reduced this effect, probably due to partial coverage of the nanotube surface by HAp. Importantly, the material was suitable for 3D printing without the need for rheology-modifying additives. The structures produced by the FDM method were characterised by good interlayer adhesion and low porosity, as confirmed by SEM images. The results of the study indicate that the developed PMMA-CNT-HAp composite is characterised by a favourable combination of biocompatibility, mechanical strength, and processability, making it a promising candidate for the production of personalised bone implants and other biomedical structures using additive technologies [149].

The results indicate that PMMA-ceramic composites are suitable for dental implants, denture bases, and prosthetic restorations, providing ‘tooth-like’ mechanical properties and low porosity, as demonstrated by studies using stereolithography and CAD/CAM methods. In the field of biomedicine, composites developed by Esmi et al., Tontowi et al., and others exhibit properties conducive to bone tissue engineering and bone defect repair, and some formulations improve osseointegration [149]. Overall, the studies confirm the potential of optimised PMMA-ceramic composites for the fabrication of patient-specific dental and bone structures with improved mechanical properties and biocompatibility.

This review highlights the important role of PMMA in the development of medical technologies for 3D printing and bone regeneration. The use of innovative ideas improves both its antibacterial properties and biocompatibility. Advances in this field offer hope for new promising solutions in medicine, due to its good printability, biocompatibility, chemical stability, and the possibility of combining it with other materials and bioactive substances [150].

An important aspect, given the high interest in this polymer, is recycling. It can be recycled in several ways, yielding different end results. The most convenient method is thermal decomposition, which can be carried out in several ways, e.g., pyrolysis or depolymerisation. This method allows for the production of high-performance methyl methacrylate (MMA) monomers. Such decomposition into monomers also makes it attractive among other polymers, where this is difficult to achieve, as it allows for reuse in the synthesis of new materials [151,152].

An important parameter is the depolymerisation efficiency, which can be close to 95% for PMMA [153]. The most common method is pyrolysis, which is carried out at a temperature of 350–500°C. An alternative is catalytic depolymerisation, i.e., using catalysts such as aluminium oxide or zeolites [154,155]. PMMA also allows for solvolysis, a process that leads to the degradation of materials using a solvent [155,156].

In summary, chemical recycling of PMMA, and in particular thermal depolymerisation in specialised reactors, is a highly efficient method of recovering high-value-added raw materials. In an era of intensive development of the circular economy and the growing importance of sustainable development strategies, these technologies represent an important direction for research and industrial implementation [155].

The presence of a ceramic phase additive to PMMA is due to improved bioactivity. The addition of apatite particles leads to increased stiffness and compressive strength, making the composite suitable for use in bone tissue engineering applications. However, excessive ceramic content can negatively affect tensile strength and elongation at break. The challenge for 3D printing will be the known problems associated with the aggregation of ceramic particles, which can cause geometric inaccuracies and an irregular internal structure of the printed element. The design of biomimetic structures must take into account the trade-offs between porosity (at the expense of strength) and the bioactivity of solid ceramic fillers. Based on literature reports, it can be assumed that the optimal amount of ceramic is between 10% and 20% by weight, which provides an ideal balance between bioactivity and printability without excessively reducing the modulus at porosity [148,157].

### 2.5. Summary

This review of four selected biopolymers, PEEK, PCL, PLA, and PMMA, reveals the diverse physicochemical and biological properties of these materials, which determine their application in biomedical engineering. Each of the polymers discussed has unique characteristics. PEEK stands out for its excellent mechanical strength and biocompatibility, PCL is known for its good biodegradability and flexibility, PLA is characterized by its easy processability and origin from renewable raw materials, while PMMA offers excellent optical transparency and chemical stability. Their properties determine specific areas of application, from implantology to tissue scaffolding to dental applications.

The most important properties are compared in Table 2, making it easier to analyze and select the right material for a specific biomedical application.

## 3. Future Prospects and Conclusions

This review summarizes recent developments in the use of thermoplastic polymers, specifically PEEK, PCL, PLA, as well as PMMA, for 3D printing in bone tissue engineering. Particular emphasis was placed on their advantages, limitations, bioactivity modifications, and application potential. Despite their great potential, there are still challenges ahead for the implementation of thermoplastic implants into the market and clinical practice. First of all, their long-term biocompatibility must be verified, taking into account the target implantation in the patient’s body. Mechanical resistance under physiological conditions, sterilizability, and meeting regulatory requirements are also not insignificant. Also, the need for standardization of printing procedures, validation of printers, surface modifications, and methods of assessing biocompatibility remains one of the main implementation issues. Controlled clinical trials are essential. Unfortunately, the number of such studies on personalized implants printed from biocompatible thermoplastics is limited, which slows down the process of transferring these technologies to the market. Perhaps a prospect for development is the use of artificial intelligence and machine learning to optimize implant design and simulate their behavior in a biological environment. In this aspect, interdisciplinary cooperation between materials engineers, biologists, and physicians is crucial to translate scientific achievements into safe and effective medical solutions.

3D printing with biocompatible thermoplastics, such as PEEK, PCL, PLA, and PMMA, described in this review, demonstrates great potential for creating personalized bone defect fillers. It provides opportunities to produce products of any personalized shape. In particular, the combination of thermoplastics with bioactive ceramic particles makes it possible to obtain materials with favorable osteoconductive properties, providing the opportunity to customize mechanical properties in the process. The observed progress in the design of new material compositions and optimization of printing parameters brings us closer to the implementation of such implants in clinical practice. However, the full implementation of their capabilities requires further research, especially in the context of long-term safety, integration with living tissue, and predictability of material degradation in the body. However, this is a promising research topic, with great application potential and an opportunity to create personalized solutions for patients with bone loss.

An emerging and particularly promising new direction in printing development is 4D printing. An advanced form of additive manufacturing that enables the creation of intelligent, stimuli-responsive implants. These structures can adapt their shape or behaviour in response to environmental stimuli, offering new opportunities for minimally invasive procedures and better integration with surrounding tissues. The use of 4D printing in bone tissue engineering could make way for the next generation of personalised, dynamic materials [165,166]. In summary, continued advances and developments in materials science, printing technology, and interdisciplinary collaboration will be essential to translate these innovations into clinical therapies.

This review provides a comprehensive synthesis of the current state of knowledge regarding the use of biocompatible thermoplastics in 3D printing, with particular emphasis on their potential for the fabrication of personalized bone implants. The analysis of material properties, processing methods, and biofunctionalization strategies offers a solid scientific foundation for further translational research. By outlining both recent advancements and key implementation barriers, this work may facilitate the acceleration of technology transfer from laboratory research to clinical application. As such, it serves as a relevant reference point for interdisciplinary research teams and clinicians involved in the development of personalized medical solutions based on 3D-printing technologies.

## Figures and Tables

**Table 1:** Mechanical properties of heat-cured PMMA [142,143,144].

PROPERTY	VALUE	UNIT
Elastic modulus (GPa)	2.6	GPa
Tensile strength	55	MPa
Vickers Hardness	20	VHN
Elongation	1–2	%
Glass transition temperature	125	°C
Flexural strength	90	MPa

**Table 2:** Mechanical properties of selected biopolymers [51,86,158,159,160,161,162,163,164].

Property	PEEK	PCL	PLA	PMMA
Elastic modulus [GPa]	2–3	0.21–0.44	3.5	2.6
Tensile strength [MPa]	70–100	4–785	59	55
Vickers Hardness [VHN]	24	7.2	25	20
Elongation [%]	50	20–1000	7	1–2
Glass transition temperature [°C]	143	(−65)– (−60)	57–60	125
Flexural strength [MPa]	140–170	12	106	90

## Data Availability

No new data were created or analyzed in this study.

## Abbreviations

The following abbreviations are used in this manuscript:

PEEK	Multidisciplinary Digital Publishing Institute
PLA	Directory of open access journals
PCL	Three-letter acronym
PMMA	Linear dichroism
HAp	Hydroxyapatite
FDM	Fused deposition modeling
β-TCP/TCP	Tricalcium phosphate
BG	Bioactive glass
SMPs	Shape memory polymers
FDA	Food and Drug Administration
ROP	Ring-opening polymerization
